# Dexmedetomidine Combined With Butorphanol or Sufentanil for the Prevention of Post-operative Nausea and Vomiting in Patients Undergoing Microvascular Decompression: A Randomized Controlled Trial

**DOI:** 10.3389/fmed.2020.583031

**Published:** 2020-10-30

**Authors:** Guangjun Xu, Jing Zhao, Zunyuan Liu, Guoying Liu, Lei Liu, Chunguang Ren, Yanchao Liu

**Affiliations:** Department of Anaesthesiology, Liaocheng People's Hospital, Liaocheng, China

**Keywords:** post-operative nausea and vomiting, microvascular decompression, butorphanol, sufentanil, dexmedetomidine

## Abstract

**Background:** Patients undergoing microvascular decompression are often accompanied with high risk of post-operative nausea and vomiting (PONV). In this study, we compare the antiemetic efficacy of butorphanol or sufentanil combined with dexmedetomidine in patients undergoing microvascular decompression.

**Methods:** Patients undergoing microvascular decompression were randomized into two groups. The primary outcome was the occurrence and severity of PONV during the 72 h after surgery. Secondary outcomes included levels of pain intensity and sedation and consumption of opioids at 1, 2, 6, 12, 24, 48, and 72 h after surgery. We also recorded the intraoperative hemodynamics, consumption of narcotic drugs, operation and anesthesia time, estimated blood loss, infusion volume and urine output, requirements of rescue antiemetics or analgesics, the satisfaction scores of patients and surgeons, complications, and length of stay.

**Results:** The overall incidence rates of nausea and vomiting during the 72 h after surgery were significantly reduced in group DB (76.00 and 44.00% in group DS vs. 54.17% and 22.92% in group DB, *P* < 0.05). Patients in group DB had a lower incidence of nausea than those in group DS at intervals of 1–6 and 6–24 h (*P* < 0.05). However, patients in group DB had a lower incidence of vomiting than those in group DS only at intervals of 1–6 h (*P* < 0.05). Similarly, the number of patients requiring rescue antiemetics was also significantly reduced in group DB compared with that in group DS at intervals of 1–6 h (*P* < 0.05). The number of patients experiencing moderate to severe PONV was comparable between the two groups during 72 h after surgery (*P* > 0.05). The consumption of opioid morphine equivalent was significantly reduced in group DB (*P* < 0.05). Compared with those in group DS, the satisfaction scores of both patients and surgeons were significantly increased in group DB (*P* < 0.05).

**Conclusion:** Butorphanol combined with dexmedetomidine could reduce early PONV and the number of patients requiring rescue antiemetics, especially at intervals of 1–6 h, while the satisfaction scores of both patients and surgeons were significantly increased.

## Background

Post-operative nausea and vomiting (PONV) is one of the most common post-operative complications in neurosurgical patients (1). It can cause electrolyte imbalance, pulmonary aspiration, elevated intracranial pressure, and delayed discharge and even result in disastrous consequences such as intracranial hemorrhage and cerebral hernia (2, 3). A previous study has reported that the incidence of PONV exceeded 50% in neurosurgical patients during the first 48 h after surgery (4). Vagal afferents of the gastrointestinal tract, chemoreceptor trigger zone in the area postrema, and the vestibular system may all have an effect on the PONV (5).

Trigeminal neuralgia (TN) is a syndrome of unilateral, paroxysmal, stabbing facial pain, originating from the trigeminal nerve and can severely affect a patient's daily lifestyle (6). Its diagnosis is extremely complicated, and careful characteristic clinical symptoms are crucial (7). The number of patients undergoing microvascular decompression (MVD), which is the best surgical modality for TN, is increasing worldwide (8). Several studies have reported that the pain-free rate was 70–80% in patients undergoing MVD at 5–10 years (9, 10). However, a previous study reported that MVD is an independent stronger risk factor for PONV even within the scope of neurosurgery (11). Though a previous study revealed that ondansetron significantly reduced PONV, the incidence of PONV was still higher than 60% within 24 h after MVD despite preventive use of ondansetron. The reason may be partly due to the operation region near the chemoreceptor trigger zone and vestibular system (12). Besides, previous study has reported that PONV may exhibit a bimodal pattern up to 48–72 h after neurosurgery (13).

Butorphanol has been widely used for musculoskeletal pain, headaches, and perioperative analgesia through the analgesia effect is only about 0.5% of sufentanil. However, there are few studies about butorphanol after neurosurgery. Dexmedetomidine (Dex), a highly selective α2-adrenergic receptor agonist, has sedative, analgesic, and anxiolytic effects (14). A recent study has also showed that sufentanil or butorphanol combined with Dex can be used safely and effectively in patients undergoing laparoscopic surgery with no increase in the incidence of adverse reactions (15). There have been no effective solutions to reduce both the incidence and severity of PONV in neurosurgery. As a result, we performed this prospective randomized clinical trial to evaluate the efficacy of butorphanol or sufentanil combined with Dex for the prevention of PONV in patients undergoing MVD.

## Methods

### Patients

All patients who underwent MVD in our hospital between November 2018 and January 2020 were recruited. This study was also approved by the ethics committee in our hospital and registered in the Chinese Clinical Trial Registry (ChiCTR1800018946). All patients or their representative have provided written informed consent.

Patients were included if they met the following criteria: diagnosed as idiopathic TN (ITN) (16) and were of American Society of Anesthesiologists (ASA) grades I and II. Patients were excluded if they have diabetes mellitus; use antiemetics or glucocorticoids; have a history of PONV or motion sickness, chemotherapy, or radiation therapy; have a body mass index (BMI) >30 kg/m^2^; have ischemic heart disease; have a history of long-term abuse of or addiction to alcohol, opioid(s), or sedative–hypnotic drug(s); are a smokers; have an allergy to opioids or Dex; have benign or malignant tumors or arteriovascular malformations confirmed with magnetic resonance imaging (MRI). All patients completed the Penn Facial Pain Scale (PFPS, formerly known as Brief Pain Inventory-Facial) on admission.

### Randomization and Blinding

A computer-generated randomization table was used to divide patients randomly into two groups by an independent anesthetist prior to surgery [group DS: Dex 4 μg/kg with sufentanil 1.0 μg/kg in the patient controlled intravenous analgesia (PCIA) pump, *n* = 50; group DB: Dex 4 μg/kg with butorphanol 0.1 mg/kg in the PCIA pump, *n* = 48]. The study drugs in the PCIA pump were prepared by an independent anesthetic nurse, while two other anesthetic nurses were responsible for recording the results of this study. The attending anesthesiologists, surgeons, anesthetic nurses in the acute pain service (APS), and patients were all blinded to this study.

### Anesthetic Management

None of the patients had received any medication before the induction of anesthesia. After patients entered the operating room, a peripheral venous access was established, and five-lead electrocardiogram, invasive arterial blood pressure, and oxygen saturation were continuously monitored by an automated system. An intravenous infusion of Dex 0.5 μg/kg was used before anesthesia induction within 15 min, followed by 0.1 mg/kg of dexamethasone, 0.3 μg/kg of sufentanil, 1–2 mg/kg of propofol, and 2.0 mg/kg of cisatracurium; we implemented trachea intubation 3 min later. Anesthesia was maintained with sevoflurane (1.5–2.5%), remifentanil (0.1–0.2 μg/kg/min), and Dex (0.4 μg/kg/h). The bispectral index was maintained between 40 and 60. The pressure of arterial carbon dioxide (PaCO_2_) was maintained at 35–40 mmHg during surgery. Both groups received 1 mg butorphanol and 5 mg tropisetron 30 min prior to the end of surgery. The concentrations of sevoflurane and remifentanil were adjusted according to both hemodynamic changes and the bispectral index. Under the premise of satisfactory depth of anesthesia, intraoperative vasoactive drugs (ephedrine, phenylephrine, urapidil, and atropine) were used to maintain hemodynamic stability.

All patients were extubated and observed in the postanesthetic care unit (PACU) until they meet Aldrete's criteria and then transferred to the functional neurosurgery ward. PCIA (Dex 4 μg/kg with sufentanil 1.0 μg/kg in the DS group and Dex 4 μg/kg with butorphanol 0.1 mg/kg in the DB group, up to a total volume of 100 ml) was programmed to deliver 1 ml bolus (lockout 8 min) with a continuous background infusion of 1 ml/h at the end of surgery. The PCIA was used for the first 72 h after surgery. Ten milligrams of metoclopramide was administered if the scores of PONV were >6 or if there was vomiting. Fifteen milligrams of ketorolac was administered if VRS scores of pain (VASm) were >3. The PCIA was stopped if hypoventilation (respiratory rate of <10 breaths per minute) or hypoxia (pulse oxygen saturation of <88% though intranasal oxygen inhalation at 5 L/min) happened.

### MVD Procedure

The operative technique was performed according to the previous studies (17, 18). Briefly, patients were placed in a lateral position after general anesthesia, and a small retromastoid craniectomy was made behind the ear after undergoing scalp nerve block (2% lidocaine combined with 1:200,000 adrenaline) before the operation in this study; then the C-shaped dura was opened. The cerebellar hemisphere was retracted gently in a superolateral-to-inferomedial direction to visualize the trigeminal nerve. The proximal part of the nerve adjacent to the brainstem was closely examined, and any compressing artery was mobilized away from the nerve. A small piece of Teflon was then interpositioned between the nerve and the artery to prevent recontact. If venous rather than arterial compression was present, the vein was coagulated and divided. When no compressing vessel was identified, internal neurolysis was performed by separating nerve fibers longitudinally. After hemostasis, the dura was closed, and the bony defect covered with gel foam before musculofascial closure in layers. Intraoperative auditory brainstem evoked response was also monitored in this surgery. Post-operative CT was performed on the day after surgery except the patient's condition deteriorated.

### Date Collection

The primary outcome was the occurrence and severity of post-operative nausea (defined as a subjectively unpleasant sensation associated with the awareness of an urge to vomit; the severity of nausea was graded using a verbal 11-point rating scale, with 0 indicating no nausea and 10 indicating the worst nausea) and vomiting (defined as a single episode of the forceful expulsion of gastric contents through the mouth) during the 72 h after surgery (19). Secondary outcomes included levels of pain intensity [visual analog scale (VAS) both at rest and with movement: 0, no pain; 10, the worst pain], sedation (LOS: recorded on a 5-point scale: 0, fully awake; 1, drowsy/closed eyes; 2, asleep/easily aroused with light tactile stimulation or a simple verbal command; 3, asleep/arousable only by strong physical stimulation; 4, unarousable), and consumption of opioids at 1, 2, 6, 12, 24, 48, and 72 h after surgery. We also recorded the intraoperative hemodynamics [recorded at the following time points: arrival at the operating room (T1); before intubation (T2); at intubation (T3); at 5 min (T4) and 10 min (T5) after intubation; at start of surgery (T6); at end of surgery (T7); at extubation (T8); and at 5 min (T9) and 10 min (T10) after arrival at the PACU], consumption of narcotic drugs, operation and anesthesia time, estimated blood loss, infusion volume and urine output, requirements of rescue antiemetics or analgesics, the satisfaction scores of patients and surgeons (11-point scale: 0, poorest; 10, excellent), complications (such as headaches, intracranial hemorrhage, wound infection, confusion, transient facial numbness, and diplopia), and length of stay.

### Statistical Analysis

In our pilot study, 49% of patients receiving Dex–sufentanil experienced vomiting during the 72 h after surgery. We considered a 27% reduction to be clinically significant; 45 patients were needed in each group at a level of 0.05 and with power of 80%. Assuming a dropout rate of 10%, we included 50 patients in each group.

The Kolmogorov–Smirnov and Levene tests were used to assess data distribution and homogeneity of variance, respectively. Continuous data were expressed as mean and standard deviation or median and interquartile range (IQR). Between-group comparisons were performed using repeated-measures analysis of variance. Mann–Whitney *U* test was used for non-normal distribution of continuous data. Categorical data were expressed as frequency and percentage and analyzed using chi-square tests or Fisher's exact tests when appropriate. A probability *P* < 0.05 was considered statistically significant. Statistical analysis was performed with SPSS for Windows version 23.0 (SPSS Inc., Chicago, IL, USA).

## Results

### Baseline Characteristics

**Figure 1:**
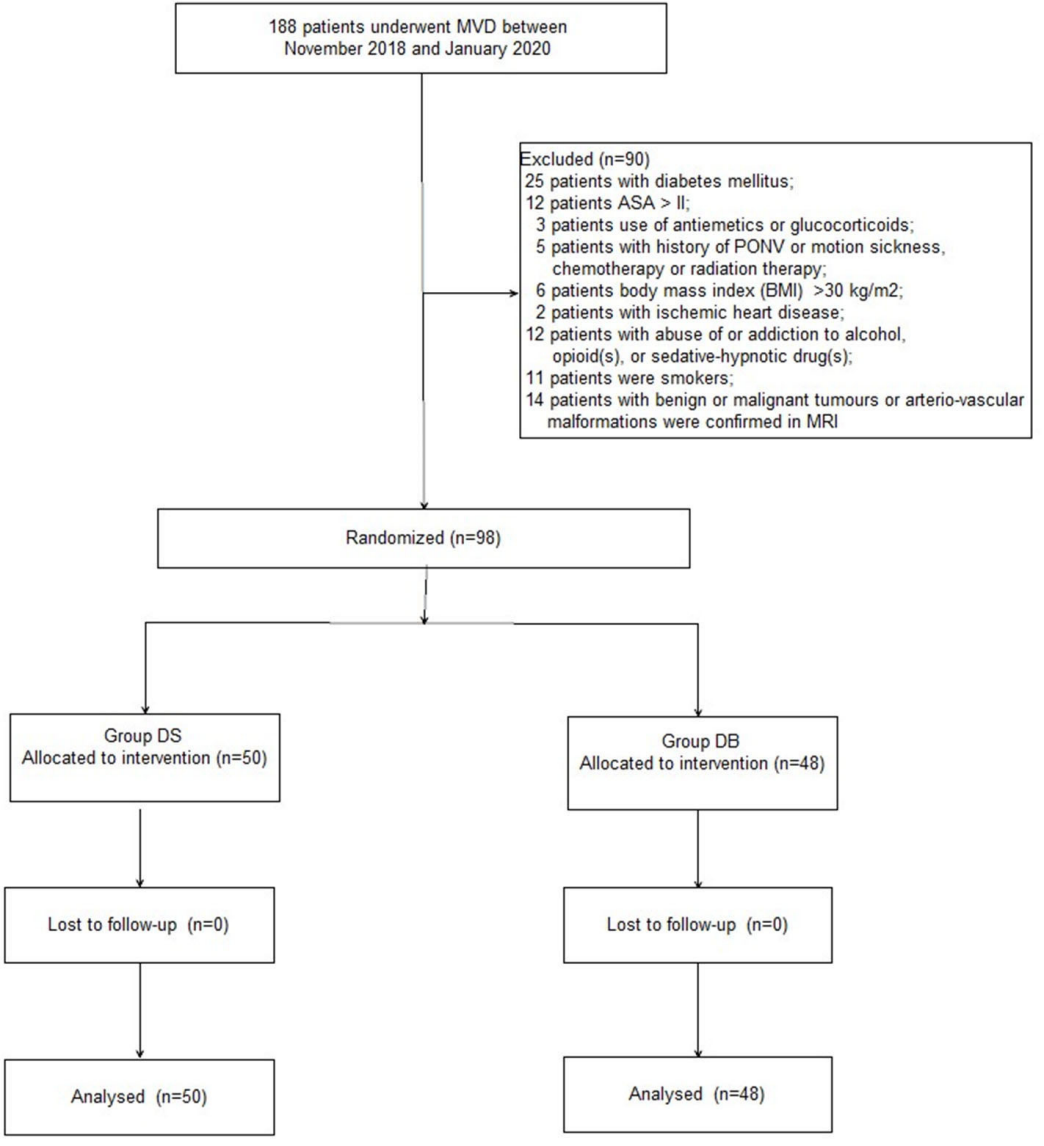
Patient enrollment flow diagram.

A CONSORT diagram was used during the enrollment of patients (Figure 1). One hundred eighty-eight patients who underwent MVD in our hospital between November 2018 and January 2020 were recruited. Ninety patients were excluded: 25 patients with diabetes mellitus; 12 patients with an ASA grade >II; 3 patients who used antiemetics or glucocorticoids; five patients with a history of PONV or motion sickness, chemotherapy, or radiation therapy; six patients with a BMI of >30 kg/m^2^; 2 patients with ischemic heart disease; 12 patients with abuse of or addiction to alcohol, opioid(s), or sedative–hypnotic drug(s); 11 patients who smoked; and 14 patients with benign or malignant tumors or arteriovascular malformations confirmed through MRI. Finally, 98 patients were included in the primary analysis and divided into two groups: 50 patients for group DS and 48 patients for group DB. Age, BMI, ASA grade, sex, comorbidity, history of TN, trigeminal nerve pain distribution, neurovascular compression, and PFPS score were all comparable between the two groups (*P* > 0.05, Table 1).

**Table 1:** Comparison of patient characteristics between the two groups.

**Variable**	**Group DS (*n* = 50)**	**Group DB (*n* = 48)**	***P*-values**
Age (years)	58.53 ± 3.89	56.72 ± 3.21	0.069.
Body weight (kg)	68.22 ± 6.03	72.55 ± 7.70	0.281.
BMI (kg·m^−2^)	24.34 ± 1.68	24.59 ± 2.11	0.517
ASA I/II (*n*)	26/24	22/26	0.542
Sex (Male/Female)	17/33	19/29	0.567
Left-sided pain*, n* (%)	24 (60.00%)	22 (45.83%)	0.830
History of TN (month)	35.43 (21.45–55.67)	37.53 (20.34–54.56)	0.188
Comorbidity, *n* (%)			0.931
Hypertension	22 (44.00%)	18 (37.50%)	
cerebral infarction	5 (10.00%)	4 (8.33%)	
Coronary heart disease	6 (12.00%)	7 (14.58%)	
Trigeminal nerve pain			0.882
distribution, *n* (%)			
V1	12 (24.00%)	13 (27.08%)	
V2	43 (86.00%)	40 (83.33%)	
V2 V3	26 (52.00%)	22 (45.83%)	
Neurovascular compression, *n* (%)			0.921
Artery	35 (70.00%)	32 (66.67%)	
Vein	9 (18.00%)	11 (22.92%)	
Artery and vein	4 (8.00%)	3 (6.25%)	
None	6 (12.00%)	5 (10.42%)	
PFPS score			0.421
General function	6.38 (5.64–6.78)	6.45 (5.61–6.85)	
facial function	7.65 (6.43–8.86)	7.32 (6.29–8.71)	

*Variables presented as mean ± SD, median (interquartile range) or number of patients n (%). BMI, body mass index; ASA, American Society of Anesthesiology; TN, Trigeminal neuralgia; PFPS, Penn Facial Pain Scale*.

### Intraoperative Variables

**Figure 2:**
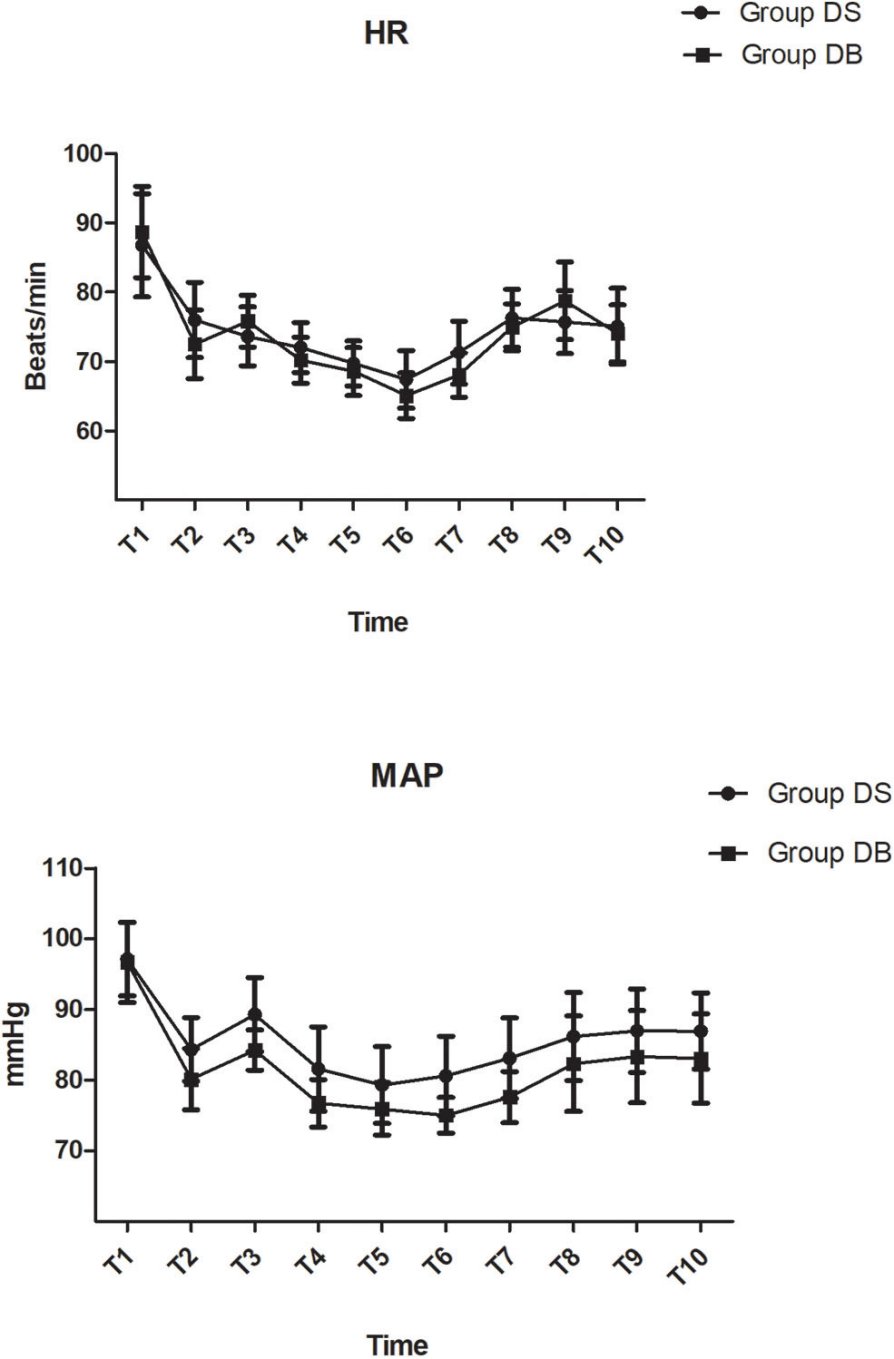
Intraoperative hemodynamic changes.

There were no significant differences between the two groups in terms of operation and anesthesia time; intraoperative hemodynamics; consumption of sevoflurane, remifentanil, Dex, and cisatracurium; estimated blood loss; infusion volume; and urine output (*P* > 0.05, Table 2, Figure 2). The number of patients using atropine, ephedrine, phenylephrine, and urapidil was also comparable between the two groups during operation (*P* > 0.05, Table 2).

**Table 2:** Comparison of intraoperative variables between the two groups.

**Variable**	**Group DS (*n* = 50)**	**Group DB (*n* = 48)**	***P*-values**
Duration of surgery (min)	163.58 (125.10–197.23)	175.57 (127.34–198.21)	0.231
Duration of anesthesia (min)	215.83 (185.99–256.83)	230.52 (196.87–270.516)	0.096
Remifentanil dosage (mg)	1.02 ± 0.29	1.12 ± 0.35	0.126
Dexmedetomidine dosage (μg)	122.38 ± 17.82	125.71 ± 23.19	0.426
Cisatracurium dosage (mg)	22.34 ± 1.89	21.83 ± 2.05	0.203
Sevoflurane (%)	1.74 (1.35–2.28)	1.59 (1.32–2.37)	0.108
Estimated blood loss (ml)	63.27 (45.38–102.74)	73.29 (52.23–109.21)	0.276
Fluids (ml)	1523.98 (683.28–2312.32)	1322.74 (836.28–2271.38)	0.075
Urine output (ml)	873.28 (462.81–1327.98)	809.72 (530.29–1529.87)	0.387
**Number of patients using vasoactive drugs**, ***n*** **(%)**			
Atropine	6 (12.00%)	5 (10.42%)	0.804
Ephedrine	4 (8.00%)	3 (6.25%)	1.000
Phenylephrine	17 (34.00%)	13 (27.08%)	0.458
Urapidil	6 (12.00%)	8 (16.67%)	0.509

*Variables presented as mean ± SD, median (interquartile range) or n (%)*.

### Post-operative Variables

**Figure 3:**
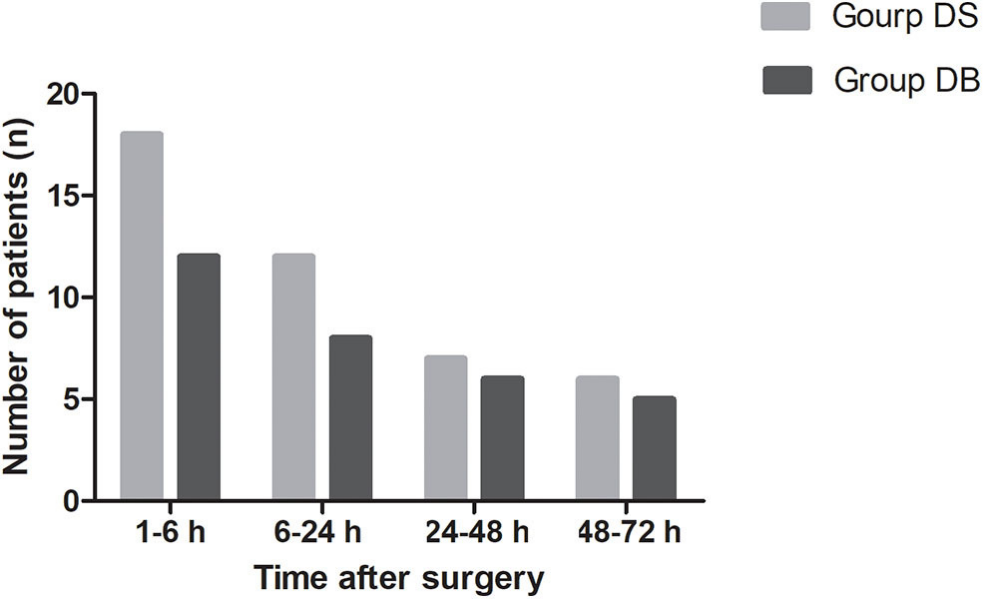
Number of patients who experienced moderate to severe PONV between the two groups during 72 h after surgery. Moderate to severe PONV: severity of nausea >3 and vomiting.

Compared with those in group DS, the overall incidence rates of nausea and vomiting during the 72 h after surgery were significantly reduced in group DB (76.00% and 44.00% in group DS vs. 54.17 and 22.92% in group DB, *P* < 0.05, Table 3). Patients in group DB had a lower incidence of nausea than patients in group DS at intervals of 1–6 and 6–24 h (*P* < 0.05, Table 3). However, patients in group DB had a lower incidence of vomiting than patients in group DS only at intervals of 1–6 h (*P* < 0.05, Table 3). Similarly, the number of patients requiring rescue antiemetics was also significantly reduced in group DB compared with group DS at intervals of 1–6 h (*P* < 0.05, Table 3). The number of patients who experienced moderate to severe PONV (severity of nausea >3 and vomiting) was comparable between the two groups during 72 h after surgery (*P* > 0.05, Figure 3).

**Table 3:** Incidence of PONV and rescued antiemetics between the two groups.

**Variable**	**Group DS (*n* = 50)**	**Group DB (*n* = 48)**	***P*-values**
Nausea, *n* (%)			
1-6 h	29 (58.00%)	18 (37.50%)^*^	0.042
6–24 h	22 (44.00%)	12 (25.00%)^*^	0.048
24–48 h	13 (26.00%)	9 (18.75%)	0.39
48–72 h	10 (20.00%)	8 (16.67%)	0.67
Vomiting, *n* (%)			
1–6 h	15 (30.00%)	8 (16.67%)^*^	0.049
6–24 h	12 (16.00%)	5 (10.42%)	0.121
24–48 h	5 (10.00%)	6 (12.50%)	0.695
48–72 h	3 (6.00%)	3 (6.25%)	0.959
Rescued antiemetics, *n* (%)			
1–6 h	22 (44.00%)	11 (22.91%)^*^	0.027
6–24 h	16 (32.00%)	9 (18.75%)	0.133
24–48 h	9 (18.00%)	7 (14.58%)	0.647
48–72 h	8 (16.00%)	5 (10.42%)	0.415

*Variables presented as number of patients n (%)*.

* *P <0.05 vs. Group DS*.

**Figure 4:**
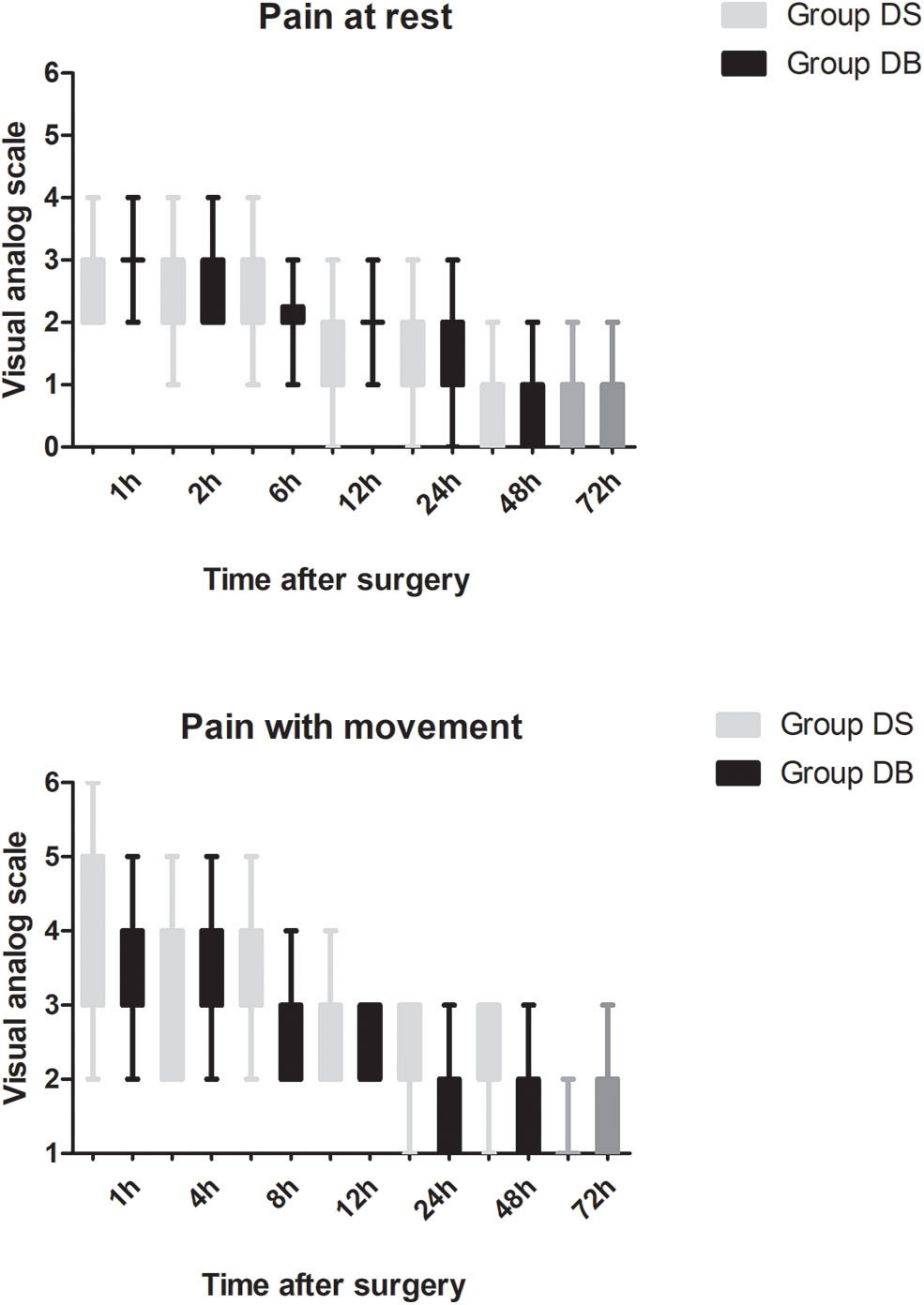
Post-operative pain intensity (at rest and with movement) between the two groups.

**Figure 5:**
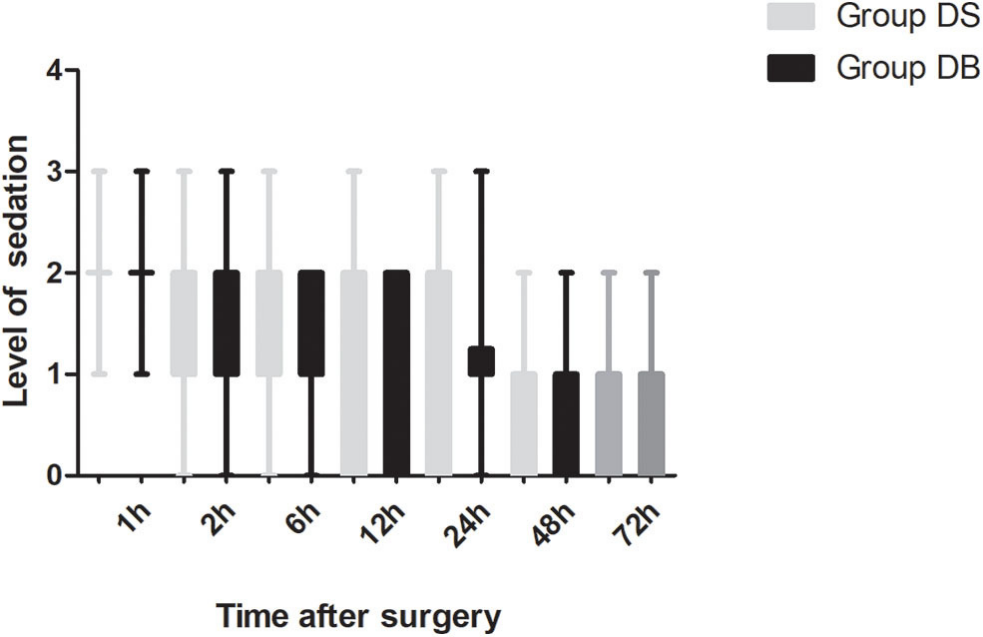
Post-operative level of sedation between the two groups.

**Figure 6:**
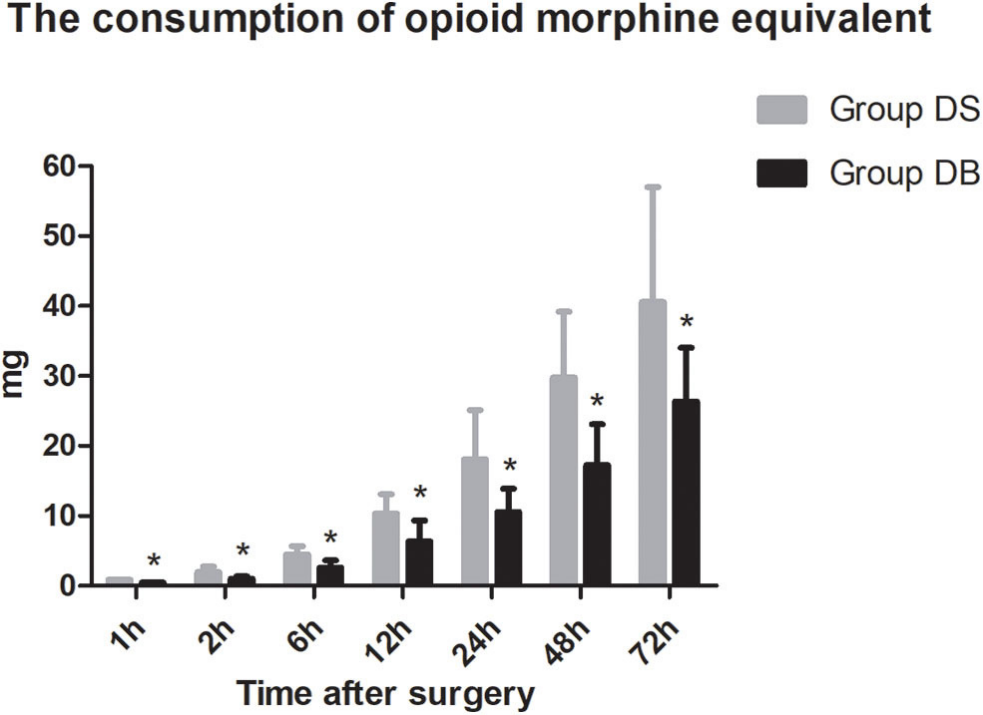
Consumption of opioid morphine equivalent between the two groups. *P < 0.05 vs. group DS.

Patients requiring rescue analgesia and length of stay were comparable between the two groups (*P* > 0.05, Table 4). Compared with group DS, the satisfaction scores of both patients and surgeons were significantly increased in group DB (*P* < 0.05, Table 4). Pain scores and LOS were not significantly different between the two groups (*P* > 0.05, Figures 4, 5). The consumption of PCIA was similar between the two groups. However, the consumption of opioid morphine equivalent was significantly reduced in group DB (*P* < 0.05, Figure 6). Complications after the surgery are summarized in Table 5. There were no mortalities in this study.

**Table 4:** Comparison of post-operative variables between the two groups.

**Variable**	**Group DS (*n* = 50)**	**Group DB (*n* = 48)**	***P*–values**
Number of rescue analgesia, *n* (%)	5 (10.00%)	8 (16.67%)	0.331
Patient satisfaction score	7.50 (6.25–8.50)	8.50 (7.25–9.50)^*^	0.021
Surgeons satisfaction score	8.00 (7.50–9.50)	8.75 (8.00–9.75)^*^	0.028
Length of stay (d)	6.45 (5.53–8.24)	6.83 (5.48–8.31)	0.398

*Variables presented as number of patients n (%) or median (interquartile range)*.

* *P <0.05 vs. Group DS*.

**Table 5:** Complications of patients undergoing MVD.

**Variable**	**Group DS (*n* = 50)**	**Group DB (*n* = 48)**	***P*–values**
Headaches	5 (10.00%)	4 (8.33%)	1.000
Dizzy	2 (4.00%)	2 (4.17%)	1.000
Transient facial numbness	3 (6.00%)	2 (4.17%)	1.000
Intracranial hemorrhage	1 (2.00%)	0 (0.00%)	1.000
Prolonged confusion	0 (0.00%)	1 (2.08%)	0.490
Cerebrospinal fluid leak	1 (2.00%)	1 (2.08%)	1.000
Diplopia	1 (2.00%)	1 (2.08%)	1.000

*Variables presented as number of patients n (%)*.

## Discussion

The results of this study indicated that butorphanol combined with Dex could reduce early PONV and the number of patients requiring rescue antiemetics, especially at intervals of 1–6 h, while the satisfaction scores of both patients and surgeons were significantly increased. At the same time, pain scores, LOS, the number of patients requiring rescue analgesia, and complications had not increased.

Although the exact etiology of PONV is unknown, female sex, non-smokers, history of PONV or motion sickness, post-operative use of opioids, and type of surgery are the most important independent risk factors for PONV (20). As a result, we excluded patients with a history of PONV or motion sickness and smokers in this study. In consideration of the same operation and without statistical difference about sex ratio in the two groups in this study, post-operative use of opioids has become the major factor of PONV. MVD, the most effective procedure in terms of long-term pain relief for patients with TN until now, has been considered as a surgical factor for PONV according to the consensus guidelines for managing PONV (21). As a result, combination of antiemetics with different mechanisms such as histamine, 5-hydroxytryptamine type 3 (5-HT3), acetylcholine, dopamine type 2, substance P, neurokinin, several opioid receptors, and other biomolecules is recommended for PONV (22). However, there has been no effective scheme to significantly reduce the PONV of patients undergoing MVD.

Opioid-based PCIA has been widely used in post-operative analgesia for its analgetic effectiveness; however, it can also associate with a number of side effects such as PONV, respiratory depression, pruritus, and urinary retention (23). A PCIA pump was used for 72 h after surgery in this study because a previous study found that air around the surgical sites may trigger nearby-area postrema and that pneumocephalus resolves by 31% per day after craniotomy, which was also a risk factor of PONV (24). Ha et al. reported that the antiemetic efficacy of ramosetron was similar to that of ondansetron and only reduced the severity of nausea between 6 and 24 h after MVD, which suggested that ramosetron or ondansetron alone may be too weak to prevent PONV in high-risk patients (25). Although administration of dexamethasone 4 mg and ondansetron 4 mg was found to decrease the incidence of PONV, this decrease was not significantly different because MVD is a high probability in PONV (12). Fabling et al. suggested using ondansetron 8 mg at the time of wound closure for adults who underwent infratentorial craniotomy. However, patients undergoing MVD still had a high frequency of nausea (26). Palonosetron, the latest 5-HT3 receptor antagonist and more effective than ramosetron, has been proven to prevent PONV during the first 24 h after surgery when administered during anesthetic induction. This may be due to the long peak concentration time and duration of action. This study has also reported that the incidence of PONV was only significantly reduced when prophylactic palonosetron and sugammadex were used together under propofol-maintained anesthesia (27). However, most of the above studies had not focused on the post-operative use of opioids.

It has been reported that Dex at 0.5 or 1.0 μg/kg effectively reduced the incidence of PONV compared with placebo. The mechanism may involve inhibiting inflammatory mediators and enhancing the antiemetic efficacies of 5-hydroxytryptamine type (5-HT) receptor antagonists and α-adrenergic receptors (28). Besides, our previous study has also supported an opioid-sparing effect as the underlying mechanism of the antiemetic effect of Dex (29). As a result, we adopt Dex as the adjuvant drug in the opioid-dominated PCIA. The incidence of PONV was significantly reduced in our study compared with the previous study. The reason may be the preventive application of 0.1 mg/kg dexamethasone during the period of anesthesia induction and 5 mg tropisetron 30 min prior to the end of surgery. We used higher doses of dexamethasone compared with the previous study for its characters of inhibition of inflammatory mediators and the hypothalamus–pituitary–adrenal axis, activation of α2-adrenoreceptors, and antiemetic sparing effect of Dex (11). Another possible explanation for the lower incidence of PONV may be the different anesthetic technique. Less muscle relaxants with lower doses of volatile agent and higher doses of Dex were used to allow recording of intraoperative auditory brainstem evoked response in our study. As a result, only a few patients undergoing MVD received neostigmine at the end of surgery, where it has been reported that reversal agents are associated with PONV (30). All patients underwent scalp nerve block with 2% lidocaine combined with 1:200,000 adrenaline before the operation in this study for the previous study has reported that local anesthesia or peripheral nerve block can contribute to reducing the amount of opioid used for post-operative analgesia (31). Another previous study reported that rebound pain is a very severe type of pain that appears when the peripheral nerve block wears off (32). We still observed this phenomenon, especially in the last 48–72 h post-operatively, despite adopting the multimodal analgesia regime in our study.

Butorphanol, a lipid-soluble narcotic agent with a strong κ-receptor agonist, weak u-receptor agonist/antagonist activity, and no obvious activity on δ-opioid receptors, has been widely used for musculoskeletal pain, headaches, and perioperative analgesia (33). However, there are few studies about the application of butorphanol during neurosurgery (34). In our study, we found that the consumption of opioid was significantly reduced in group DB, which may also contribute to the lower PONV and higher satisfaction scores of both patients and surgeons. Moderate sedation of patients following neurosurgery is necessary to maintain hemodynamic stability, provide sufficient analgesia, and reduce anxiety without interfering with the evaluation of the conscious state (35). As a result, the level of sedation was maintained at 1-2 in most patients during the first 12 h after surgery. Dex combined with butorphanol after neurosurgery may cause excessive sedation. However, no excessive sedation was observed during post-operative PCIA in our study. This may be due to less dosage used than the previous study (15). At the same time, pain scores and the number of patients requiring rescue analgesia had not increased. Further studies are required to establish the effect–dose balance between optimal post-operative analgesia and PONV in the Dex–butorphanol analgesic regimen.

Consistent with previous report, the age of onset for most idiopathic cases is between 50 and 60 years, and there is a higher proportion of females in our study (36). In our study, there were no mortalities or life-threatening morbidities in each group. There were still 12 vs. 10.42% patients without compressing vessel during surgery in our study, and internal neurolysis was performed by dividing the nerve. The result was similar to previous studies (7% of endoscopic MVD vs. 11% of microscopic MVD). Though endoscopic MVD has the benefit of improved visualization during surgery, the disadvantages are obvious such as having a 2D view, occupying space by itself, and generating heat that could potentially harm adjacent structures (37). The other frequently reported complications of MVD include headaches, diplopia, facial weakness, intracranial infarct/hematoma, and cerebrospinal fluid (CSF) leak. However, most of these complications are mild and transient. The incidence of complications in our study is lower than that in previous studies, the reasons for which may be the careful surgical technique (move the compressed artery distally and attach it to the dura mater using a polytetrafluoroethylene sheet, preserve the superior petrosal vein, and try to not use the retractor), absolute hemostasis, immaculate wound closure, and use of intraoperative auditory brainstem evoked response (38). It should be noted that one patient in group DB has facial paralysis immediately after surgery, which failed to resolve until discharge. However, no changes in the intraoperative auditory brainstem evoked response were observed.

There are some limitations in this study. First, we adopted the volatile-maintained anesthesia in this study due to low prices. However, a previous study reported that the incidence of PONV may be lower under propofol-maintained anesthesia in patients undergoing craniotomy (39). Second, we have no long-term follow-up about the effect of operation in this study. Third, we only reported results of patients with ITN, which are not applicable for patients with atypical and recurrent TN. Fourth, PONV decreased in the last 72 h though PCIA doses were doubled in both groups. The reasons may be complex and need further study to clarify. Finally, the result of this study only represented the practice of our center and therefore may lack generalizability to other hospitals.

In conclusion, butorphanol combined with Dex could reduce early PONV and the number of patients requiring rescue antiemetics, especially at intervals of 1–6 h, while the satisfaction scores of both patients and surgeons were significantly increased. At the same time, pain scores, LOS, the number of patients requiring rescue analgesia, and complications had not increased.

## Data Availability Statement

The original contributions presented in the study are included in the article/supplementary material, further inquiries can be directed to the corresponding author/s.

## Ethics Statement

The studies involving human participants were reviewed and approved by the Ethics Committee in Liaocheng People's Hospital and registered in the Chinese Clinical Trial Registry (ChiCTR1800018946). The patients/participants provided their written informed consent to participate in this study.

## Author's Note

The authors intend to share participants' data collected during the trial. This includes, study protocol, statistical analysis plan, informed consent forms, and clinical study report. It will be available based on the request of investigators whose proposed use of the data has been approved by an independent review committee by sending an email to the corresponding author. Data are available immediately after publication and with no end date.

## Author Contributions

GX, JZ, ZL, and LL conceived and designed the trial. GL and LL collected the date. CR and JZ analyzed the date. GX, JZ, ZL, and YL wrote the manuscript. All authors contributed to the article and approved the submitted version.

## Conflict of Interest

The authors declare that the research was conducted in the absence of any commercial or financial relationships that could be construed as a potential conflict of interest.
